# Comparing 3DQRS and VCG approaches for ECG QRS detection within 1.5T, 3T and 7T MRI

**DOI:** 10.1186/1532-429X-16-S1-P148

**Published:** 2014-01-16

**Authors:** Thomas S Gregory, Ehud J Schmidt, Shelley H Zhang, Zion T Tse

**Affiliations:** 1 College of Engineering, University of Georgia, Athens, Georgia, USA; 2 Brigham and Women's Hospital, Boston, Massachusetts, USA

## Background

Electrocardiograms (ECGs) obtained within High-field MRIs are distorted due to the Magneto-hydrodynamic (MHD) effect. Blood plasma electrolytes ejected into the aorta during early systole interact with the strong magnetic field of the MR scanner to produce a MHD-induced voltage (VMHD) [1]. The VMHD overlay on ECG traces can result in intermittent QRS detection. Vectorcardiogram (VCG) based gating approaches have been conventionally adapted in most MRI scanners [2], but may fail at high field strengths [3]. Recently, a multiple ECG channel cross-correlation based algorithm, 3DQRS, has been developed to provide increased sensitivity levels in these environments [4]. The 3DQRS approach constructs a 3-D ECG representation, where the third dimension, in addition to voltage and time, is deemed a channels axis, formed from concurrent viewing of the precordial leads V1-V6. This study quantitatively compares 3DQRS and VCG approaches at a variety of MRI field strengths to assess the robustness of these methods.

## Methods

**Figure 1:**
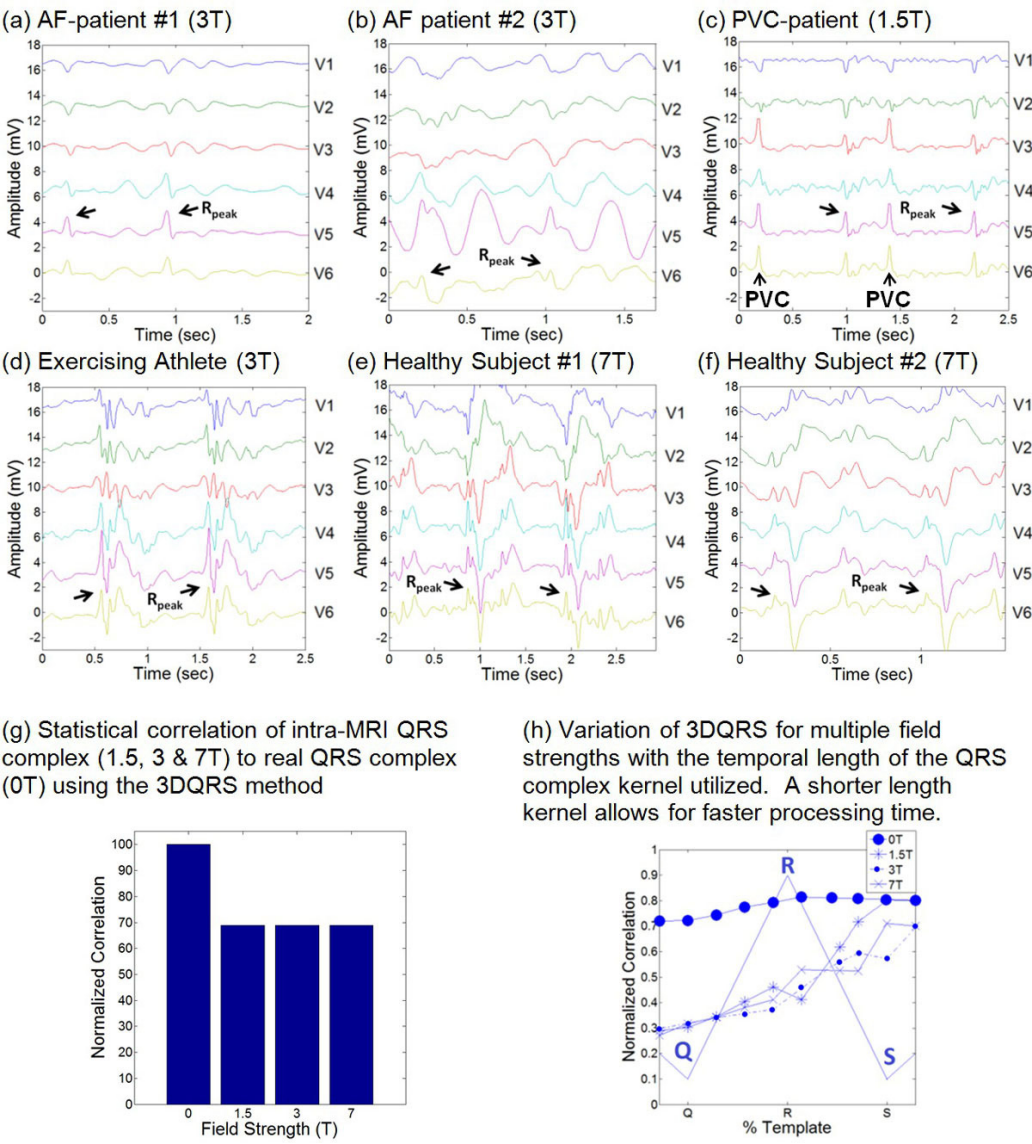
**(a-f) Representative 2 cardiac cycles of the precordial leads V1-V6 for patients and volunteers at various field strengths ("Rpeak" denotes the R-wave peak positions)**. (g-h) 3DQRS performance evaluation, showing its performance at various field strengths.

12-lead ECG data was recorded using a prototype MRI-conditional 12-lead ECG recorder [5] from 2 Premature Ventricular Contraction (PVC) patients, 2 Atrial Fibrillation (AF) patients, and a healthy exercising athlete at 1.5T and 3T [4]. A Halter recorder was used in 2 healthy volunteers at 7T [6] (Figure 1a-f). QRS detection was performed using a VCG-based approach (V1-V6, I, II) [2] and 3DQRS (V1-V6) using standard 12-lead ECG chest positioning [4]. Assessments of 3DQRS robustness relative to variations in field strength and cross-correlation kernel temporal length were performed (Figure 1g-h). False Positive (FP) and False Negative (FN) counts were recorded (total of 1,262 beats) in order to assess the sensitivity for QRS detection for each method at 1.5T, 3T, and 7T.

## Results

Table 1 shows the gating results for 3DQRS and VCG at 1.5T, 3T, and 7T in various subjects. 3DQRS subject-averaged accuracy levels in QRS detection (False Negative), relative to VCG, were: 1.5T (100% vs. 96.6%), 3T (98.1% vs. 87%), 7T (96% vs. 71.2%). In PVC patients at 1.5T, 3DQRS separated between the SR and PVC beats with 100% accuracy, whereas VCG falsely detected PVC beats, which were of similar length and magnitude to the sinus rhythm beats, with only 37.3% accuracy (Table 1).

**Table 1:** Results of 3DQRS and VCG Efficacy Tests at 1.5T, 3T, and 7T

--		3DQRS		VCG-based		Marked
		
		False Negative	False Positive	False Negative	False Positive	Total Beats
1.5T	PVC at 1.5T	0	0	2	37	59
	
	Percent of Total	0.0%	0.0%	3.4%	62.7%	--

3T	--	--	--	--	--	--
	
	AF-Diagnosed #1 at 3T	3	1	3	6	45
	
	AF-Diagnosed #2 at 3T	1	1	34	18	169
	
	Exercising Athlete at 3T	2	2	41	46	316
	
	Total Count	6	4	41	46	316
	
	Percent of Total	1.9%	1.3%	13.0%	14.6%	--

7T	--	--	--	--	--	--
	
	Healthy Subject #1 at 7T	6	6	175	156	382
	
	Healthy Subject #2 at 7T	29	30	80	56	505
	
	Total Count	35	36	255	212	887
	
	Percent of Total	4.0%	4.1%	28.8%	23.9%	--

## Conclusions

The 3DQRS method represented a higher sensitivity in QRS detection than the VCG based approach, which resulted in decreased error levels in high field MRI.

## Funding

NIH U41-RR019703, NIH R03 EB013873-01A1, SBIR-1 R43 HL110427-01.
